# A Neuro-Musculo-Skeletal Model for Insects With Data-driven Optimization

**DOI:** 10.1038/s41598-018-20093-x

**Published:** 2018-02-01

**Authors:** Shihui Guo, Juncong Lin, Toni Wöhrl, Minghong Liao

**Affiliations:** 1 Xiamen University, Software School, Xiamen, 361005 P.R. China; 2 Friedrich Schiller University Jena, Motion Science, Jena, D-07749 Germany

## Abstract

Simulating the locomotion of insects is beneficial to many areas such as experimental biology, computer animation and robotics. This work proposes a neuro-musculo-skeletal model, which integrates the biological inspirations from real insects and reproduces the gait pattern on virtual insects. The neural system is a network of spiking neurons, whose spiking patterns are controlled by the input currents. The spiking pattern provides a uniform representation of sensory information, high-level commands and control strategy. The muscle models are designed following the characteristic Hill-type muscle with customized force-length and force-velocity relationships. The model parameters, including both the neural and muscular components, are optimized via an approach of evolutionary optimization, with the data captured from real insects. The results show that the simulated gait pattern, including joint trajectories, matches the experimental data collected from real ants walking in the free mode. The simulated character is capable of moving at different directions and traversing uneven terrains.

## Introduction

Insects are the largest group of species on the planet^1^. Their success is critically dependent on their mobility. Although insects are relatively small in terms of both body size and neural system, their capability of handling complex environment outperforms most artificial robots. Simulating the locomotion of virtual insects are of great interest to researchers from various fields including experimental biology, computer animation and robotics.

Experimental biologists used the simulation model to repeat experiments which otherwise would be impossible to conduct on the vivo subjects, in order to illuminate the underlying principles of their locomotory mechanisms. The early attempt is *Walknet*^2^, which simulated the movement of walking stick insects. The follow-up works extended the standard *Walknet*, which only simulated the gait in a procedurally-kinematic fashion, to advanced components including searching for foot placements^3^ and dynamics simulation^4^. Other studies simulated the gait pattern of cockroaches by incorporating rudimentary motor-neuron activation and agonist/antagonist Hill-type muscle pairs^5–7^ to analyze the gait stability of cockroach in standard and perturbed events. However, in this model the gait pattern is limited to the horizontal plane and excludes the effect of gravity. A neuron-mechanical model of a cockroach is further proposed to simulate the individual and population behaviours of real neurons^8^. However the parameters in this complex model is hand-tuned and do not allow fast-extending the skill repertoire of virtual insects. Our work uses the evolutionary algorithm to automatically set the model parameters and avoids the process of manual efforts.

Researchers in the field of computer animation target at the problem of reproducing the mobile capability of real insects on their virtual representation. Virtual insects are widely used in graphical applications, including video games, movies and virtual/augmented reality etc. In contrast to the large collection of existing works in biped simulation, the field of insect simulation is less explored. Previously researchers set up a specialized system of synchronized cameras to capture and synthesis insect motion^9^. However this method requires manual post-processing and specialized hardware. Researchers also tackled this problem by procedurally but kinematically modelling the gait of multi-legged creatures^10^. By carefully selecting the foot placement, this method is able to adapt to different terrains and recover from perturbations. Due to the lack of a fully actuated model, the motion synthesized in this way is not physically-plausible and cannot take full advantage of the information from the interaction with the environment. Previous methods^11,12^ modelled the controller of virtual insects as a network of nonlinear oscillators and tuned the parameters of the network via the optimization strategy of Covariance Matrix Adaptation (CMA). The network was designed to mimic the Central Pattern Generator, which was found in real insects and in charge of their gait pattern. Researchers also modelled and animated virtual myriapoda by developing a hybrid animation system that combines kinematic and dynamic simulation^13^. While the body was driven by both rigid body simulation (for hard skeleton) and finite element method (for soft interlinks), the leg movements were synthesized in a kinematic fashion.

Robotics researchers used the simulation framework to speed up the process of robot design and control. A representative hexapod robot, *RHex*^14^, was designed to perform the double-tripod gait similar to the gait pattern observed in hexapod insects. This gait pattern allowed inherent locomotion stability. The open-loop controller was further improved by the introduction of *Central Pattern Generator* (CPG). The biological evidences show that the CPG is responsible for producing the basic gait pattern of real animals^15^. Bio-inspired robots, such as walking salamander^16^, swimming fish^17^ and flying bird^18^, are controlled by mimicking the machinery of CPG. More details can be found in the review paper on applications of CPG for locomotion control in animals and robots^19^. However, existing models of CPG are generally constructed as a network of nonlinear oscillators, rather than spiking neurons. The advantage of using nonlinear oscillator is the inherent convergence to limit cycle or point attraction for most oscillator models, thus capable of endogenously generating the gait pattern without the high-level commands. However, the challenge from oscillator-based networks is how to connect the sensory information/high-level commands with the low-level control strategy. Previous method^11^ used a precomputed look-up table. This inevitably creates a large discretized look-up table. In comparison, real organisms, including insects, use spiking neurons for the general purposes of environmental sensory, information transmission, decision-making and muscle actuation. Such observation inspires this work to explore the solution of using spiking neuron, as the alternative building block to nonlinear oscillator, to construct the neural system.

Building a simulation model to match its real equivalence is a non-trivial task. On one hand, a complex model may introduce too many parameters, which could be impossible to tune by hand. On the other hand, a simplified model may not capture the important details of the model and thus reduce the credibility and capability of the virtual model. In this work, we propose a neuro-musculo-skeletal model for virtual insects (using specifically the ant as an example) and an automatic solution to the problem of parameter optimization. More specifically:

- We propose a uniform encoding of sensory information, high-level commands and control strategy, with the spiking patterns of the neural system. Such an encoding paradigm provides the equivalent representation as real organism and shows the potential of using spiking neurons as the building block for accomplishing general tasks. The network also provides a continuous mapping from the sensory information/high-level commands to low-level control, in contrast to the discretized mapping in previous work^11^.
- We use the evolutionary algorithm to automatically find the optimal values for both the controller and muscle parameters. Given the high dimensions of the model state spaces, we resolve the challenge by dividing the optimization into a two-stage procedure. The first stage optimizes the parameters of controller and muscles for the standard gait of walking straight forward, while the second stage progressively extends the skill repertoire by considering curve trajectories and uneven terrain.
- We integrate the prior information (foot trajectories) of real ants during the optimization process via the evolution algorithm. The introduction of ground-truth data from real ants allows the quantitative comparison between the proposed model in this work and existing models^11^. The result shows that the trajectories generated from this model presents smaller deviations from the ground-truth data.

## Method Overview

**Figure 1:**
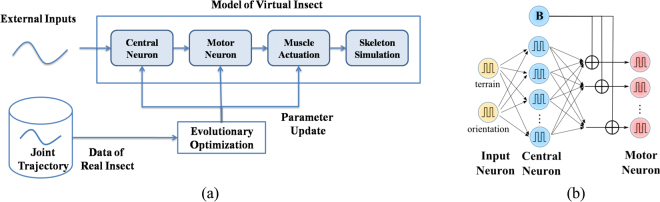
(**a**) Algorithm flowchart. (**b**) Block diagram of neural system.

The simulation framework is composed of three parts: the spiking-neuron controller, the muscle actuation and the skeleton. The spiking-neuron controller receives the external inputs (sensory information and high-level commands) and sends out a series of spiking trains during each motion cycle and activates the muscle contraction. Each skeleton joint is connected with a pair of antagonistic muscles, generating the appropriate torques and resulting in the forward movements of the virtual insect. The parameters of the neuron controller and muscle actuation are updated with the technique of evolutionary algorithm, with the captured data of real insects. The overall algorithm of our method is presented in Fig. 1(a).

### Data availability

All data generated or analysed during this study are included in this published article (and its Supplementary Information files).

## Neuro-Musculo-Skeletal Model

### Neuro-controller Model

In real insects, spiking pattern of the neural system are used for the general purposes of information transmission and processing. The controller in the proposed model is modelled as a network of spiking neurons. The unit model is based on the model proposed in the work by Izhikevich^20^. The model is endowed with both biologically plausibility of Hodgkin–Huxley-type dynamics and computational efficiency of integrate-and-fire neurons.

The equation for the spiking neuron model is:

1 $$v^{\prime} =0.04{v}^{2}+5v+140-u+I$$

2 $$u^{\prime} =a(bv-u)$$

with the auxiliary after-spike resetting:

3 $${\rm{if}}\,v\ge {\rm{30}}\,{\rm{mV}},then\{\begin{array}{ll}v\,\leftarrow  & c\\ u\,\leftarrow  & u+d\end{array}$$

*v* represents the membrane potential of the neuron. *u* represents the activation of *K*^+^ ionic currents and inactivation of *Na*^+^ ionic currents, providing negative feedback to *v*. This model has four dimensionless parameters: *a*, *b*, *c*, *d*. The model is capable of generating multiple spiking patterns, including regular spiking, intrinsically spiking, chattering and so on. We here choose the most common type of spiking pattern (regular spiking) and its corresponding parameters are: *a* = 0.02, *b* = 0.2, *c* =−65 *mV*, *d* = 8.

One novelty of this work is to provide a uniform encoding of both sensory information, high-level commands and control strategy. We choose specifically the slope angle as the example of the sensory information and the action of turning as the example of high-level commands. For the slope angle *θ*_*s*_ or the target orientation for next stride *θ*_*t*_, the input currents *I* are defined as

4 $${I}_{sensor}=\frac{{\theta }_{s}}{{\theta }_{max}^{s}}{I}_{max}^{s}$$

5 $${I}_{target}=\frac{{\theta }_{t}}{{\theta }_{max}^{t}}{I}_{max}^{t}$$

${\theta }_{max}^{s},{\theta }_{max}^{t}$ are the maximum slope angle and target orientation. ${I}_{max}^{s},{I}_{max}^{t}$ are the corresponding maximum input currents. Here both variables are set to 20 *mA*.

The input currents *I*_*sensor*_, *I*_*target*_ are fed into an input layer of spiking neurons. The input layer is connected with a central layer of 16 spiking neurons, which are then fully-connected to the motor neuron. The output from the central layer is added with a bias component (denoted in blue in Fig. 1(b)), which generates the standard gait pattern of walking straight forward. This constructs a neural network with spiking neurons (Fig. 1(b)). The synaptic connection weights between the neuron layers determine how the external information adjusts the control signals from the bias neurons and eventually modifies the standard gait pattern.

For the control strategy, each muscle is activated by a unit neuron model, which produces the overall spiking pattern of the neuron group activating the single muscle. Each joint is actuated by a pair of agonist/antagonist muscles, each of which is individually activated by one neuron controller. The separate control of individual muscles mimics the distributed hierarchy of the neural control in real insect.

### Muscular Model

The typical Hill-type muscle model is introduced as the building block for the actuation system of the virtual insect^21,22^. To simulate a virtual muscle, we need to model the dynamics of activation and contraction.

The activation process converts the spiking trains from the neural system into the activation signal on the muscle. This is modelled as a first-order differential equation:

6 $${a}_{t+1}=({v}_{t}-{a}_{t}){\rm{\Delta }}t+{a}_{t}$$

where *a*_*t*_ is the activation signal at time *t*, *v*_*t*_ is the excitation from the motor neuron (Equation 2). Δ*t* is the stepsize.

To simulate the contraction dynamics of a Hill-type muscle, we need to model two main features: the force—length and force—velocity relationships. The force—length (FL) part models the function of both active and passive forces in terms of the muscle length, and the force—velocity (FV) part describes how the contraction force varies with respect to the muscle’s contraction velocity.

7 $${F}_{CE}={F}_{max}\,\ast \,{a}_{t}\,\ast \,{F}_{L}\,\ast \,{F}_{V}$$

8 $${F}_{L}=exp(-{|\frac{{L}_{ce}^{\beta }-1}{\omega }|}^{\rho })$$

9 $${F}_{V}=(\begin{array}{ll}({V}_{max}-{V}_{ce})/[{V}_{max}+({c}_{V0}+{c}_{V1}{L}_{ce}){V}_{ce}], & {\rm{if}}\,{V}_{ce}\le 0\\ \left[{b}_{V}-({a}_{V0}+{a}_{V1}{L}_{ce}+{a}_{V2}{L}_{ce}^{2}){V}_{ce}\right]/({b}_{V}+{V}_{ce}), & {\rm{if}}\,{V}_{ce}\, > \,0\end{array},$$

where *a*_*t*_ is the activation signal from Equation 6. The variables *L*_*ce*_, *V*_*ce*_ are the length and contraction velocity of the muscle. To simplify the model, we set *L*_*ce*_ = 1 in the equation of *F*_*v*_, which means that the change of muscle length is ignored when computing the force-velocity relationship. This assumption simplifies the model parameters as: *c*_*V*_ = *c*_*V*0_ + *c*_*V*1_, *a*_*V*_ = *a*_*V*0_ + *a*_*V*1_ + *a*_*V*2_. The parameters Ω = (*F*_*max*_, *β*, *ρ*, *ω*, *V*_*max*_, *c*_*V*_, *b*_*V*_, *a*_*V*_) are muscle-specific and determine the overall performance of a muscle.

Existing works normally find the appropriate parameters using the collected data from vivo subjects^23^. However the experimental data are limited and may not be extended to different insects, or even different muscles on a single insect. Given the difference of the neural system between real and virtual insects, it is not of significance to follow the exact parameter values of real insects. Given the complicated model of nonlinear muscle, together with the parameters of neural system, we expect a high dimension of control space. It is challenging to reach an optimal solution for such optimization problem. In such circumstances, we propose the evolutionary algorithm to optimize the parameters of the controller and muscles simultaneously.

### Skeletal Model

**Figure 2:**
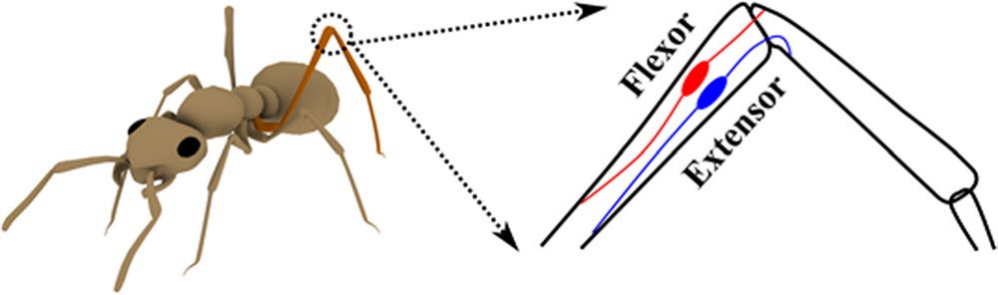
Muscle attachment of the femur-tibia joint on a virtual ant.

During locomotion, an insect must solve essentially the same problem as a vertebrate although it has an external, rather than an internal skeleton. This is reflected in how the muscles are connected to the skeleton (Fig. 2). For the skeletal model, we follow the implementation from existing work^11^ and model the virtual insect as a hierarchy of connected rigid body.

The main body is constructed as a set of three rigid bodies: head, thorax and abdomen, connected by two joints (head-thorax and thorax-abdomen). Each leg is actuated with three selected DOF: trunk-coxa, trochanter-femur and femur-tibia. Movement about the single joints and the resulting stepping patterns are generated by the activity of antagonistic muscle pairs. In the stick insect, the three major muscle pairs of a leg are the protractor and retractor coxae, the levator and depressor trochanteris, and the flexor and extensor tibiae. The protractor and retractor move the coxa, and thereby the leg, forward and backward. The levator and depressor move the femur up and down. The flexor flexes, and the extensor extends the tibia about the femur-tibia joint.

Each body segment is modelled as cylinder with its length listed in Table 1. The radius of the cylinder is set to uniform (0.2 *mm*) across all leg segments. The data are extracted from the anatomy of real ants. During the locomotion cycle, legs in stance mode are dynamically actuated and legs in swing mode are kinematically animated. This is supported by biologically evidences that each leg only occupy a small proportion of body weight (5%) (Table 2), thus do not pose significant impact on overall body movements.

**Table 1:** Length of body segments. Unit: millimeter (*mm*).

	Coxa	Femur	Tibia	Tarsus
Front	0.25	2.49	2.33	2.37
Middle	0.25	2.82	2.74	3.13
Back	0.25	3.48	3.40	3.80

**Table 2:** Mass distribution of body segments. The dimensions of each part are the lengths along *x*, *y*, *z* axes.

	Dimension (*mm*)	Mass (*mg*)	Mass Proportion (%)
Head	2.67*2.20*0.50	5.23	21.75
Thorax	3.07*1.01*0.50	3.87	16.09
Abdomen	3.43*2.46*0.50	11.35	47.19
Front Leg	0.2*0.2*7.44	0.60	5.00
Middle Leg	0.2*0.2*8.94	0.50	4.12
Back Leg	0.2*0.2*10.93	0.70	5.82

### Real animal data

**Figure 3:**
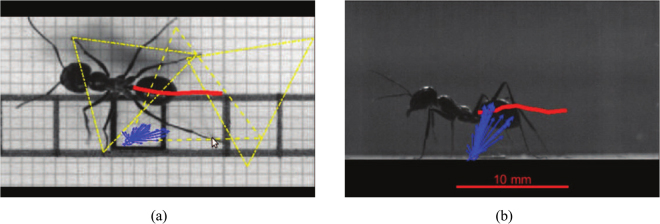
Video screenshots of real ant locomotion. The red curve denotes the trajectory of body center, the yellow triangle represents the supporting triangle formulated by touchdown positions of stance feet while the blue arrows denotes the force vectors at the stance foot. (**a**) dorsal view. (**b**) lateral view.

Real animal data on ground reaction forces and kinematics were obtained from a previous study on the leg functions of desert ants Cataglyphis fortis^24,25^. The motion of the walking ants in an enclosed chamber was captured by a Photron Fastcam SA3 (San Diego, CA, USA) camera at a frequency of 500 Hz from the lateral and the dorsal view (Fig. 3). Their ground forces were recorded with a 4 × 4 mm force platform of resolvable forces *F*_*x*_ = 5.4 *μN*, *F*_*y*_ = 2.9 *μN* and *F*_*z*_ = 10.8 *μN* and natural frequencies of *f*_*x*_ = 380 *Hz*, *f*_*y*_ = 279 *Hz* and *f*_*z*_ = 201 *Hz*^26^. The signals were amplified by a data acquisition system (MGCplus, Hottinger Baldwin Messtechnik, Darmstadt, Germany) and recorded with a sampling frequency of 1200 Hz. After each recording, the body mass was measured with an analytical balance of ±0.2 *mg* reproducibility (readout = ±0.1 *mg*, ABS 80-4, Kern & Sohn, Germany). A total of 274 trials with ground force measurements and video sequences were recorded for level locomotion. Trials in which the ants stopped, changed direction or touched the force platform with their gasters were excluded from the dataset. Subsequently, the first 15 records for each set of legs (front legs, middle legs, hind legs) were selected for data analysis resulting in 45 different strides. Besides the petiole, the head-thorax joint and the claws, the trajectories of the femur-tibia joint were additionally tracked for this study with Digitizing Tools 20160711^27^ and MATLAB R20015b (The MathWorks, Natick, MA, USA). To further model the joint torques, body parts such as the head, the thorax–petiole-coxae, the gaster and the femur-tibia-tarsi (legs) were weighed and photographed for 15 randomly selected ants.

## Optimization Algorithm

Previous paragraphs explain the hierarchy of our simulation framework. How to find the appropriate values for both the controller and muscle parameters is key to achieve stable performance of the locomotion. We here use the algorithm of Covariance Matrix Adaptation (CMA) to optimize the parameters. CMA is an evolutionary algorithm that is designed to solve nonlinear non-convex optimization problem. Existing works have applied this method to design the locomotion controller for virtual characters^11,12,28^.

The optimization is initialized with the mean vector **m** and the standard deviation vector *σ*_*cma*_. For each new generation, *λ* offspring individuals are sampled with a normal distribution:

10 $${{\bf{x}}}_{{\bf{i}}}={\bf{m}}+\sigma N\mathrm{(0},{\bf{C}}),{\bf{for}}\,{\bf{i}}=1,\cdots ,\lambda $$

The mean vector **m** represents the favourite solution from last generation. The covariance matrix **C** determines the shape of distribution ellipsoid. This covariance matrix is updated so that to increase the likelihood of successful individuals.

All individuals are evaluated and ranked by a fitness function *f*(**x**_**i**_). *μ* individuals with the lowest fitness score are selected and named as the group of elites. The new mean of the next generation is updated from a weighted sum of these *μ* elites. The covariance matrix is updated similarly as a combination of covariance from previous generation. By iteratively repeating this process, the samples are expected to converge to the optimal solution.

In the model proposed in this work, the character has 3 DOFs (3 pairs of muscles) for each leg, and the dimensions of the neuron and muscle parameters are 3 and 8 respectively for each neuron-muscle pair. The symmetry between left and right legs first reduces the dimensions of motor and muscle parameters to half. Together with the synaptic weights in the central neural system, the total dimension of parameters is 806. This far exceeds the dimensions of parameter space in existing works^11,12,28^ and the capability of the algorithm as declared by the author^29^. To optimize in the parameter space with such complexity, we resolve this challenge with a two-stage strategy with the algorithm of Covariance Matrix Adaptation (CMA). First, we optimize the input currents *I*_*s*_ to the motor neurons and muscle parameters for the standard case of walking straight forward. Next, we optimize the offset currents from the central neurons, which are added to the standard case *I*_*s*_ and generate the versatile motor skills including walking on curve trajectories and uneven terrains.

### Objective Function for Stage I

The design of the objective function is critical in selecting the elites out of the whole population and optimizing the parameters via generations of evolution. The objective functions at Stage I are designed to consider three goals simultaneously.

The first is to move forward at a predetermined velocity ν^*^ of body center (a vector pointing straight forward):

11 $${f}_{v}=\sum _{k}{(\nu -{\nu }^{\ast })}^{2}$$

where *ν* is the actual moving velocity of body center. The subscript *k* sums up all the simulation steps. The velocity is three dimensions (x, y, z) thus this objective function also penalizes the case when the body falls down or deviates from the forward direction.

The second is to match the foot trajectory **p**_*f*_ with the captured trajectory ${{\bf{p}}}_{f}^{\ast }$ from the real insect:

12 $${f}_{t}=\sum _{k}{({{\bf{p}}}_{f}-{{\bf{p}}}_{f}^{\ast })}^{2}$$

By minimizing this objective component, we expect to reproduce the similar gait pattern as the real insects. Ants typically demonstrate the double-tripod gait^11,30^. The intra-leg coordination between joints on the same leg is achieved by matching the trajectory of the particular leg, while the inter-leg coordination between different legs is enforced by the spatial-temporal information embedded in the trajectories of all legs on real ants. The use of motion trajectories allows the compact representation of the desired gait pattern, as part of the optimization goal.

The third is to minimize the energy consumption of the neural system, that is to use as little current input *I* as possible:

13 $${f}_{s}=\sum _{k}{I}^{2}$$

This objective is biologically valid since the individuals use less energy for neural control gains advantages during the process of evolution.

The final objective function is defined as:

14 $$f={\omega }_{s}\,{f}_{s}+{\omega }_{v}\,{f}_{v}+{\omega }_{t}\,{f}_{t}$$

*ω*_*s*_ = 1.0, *ω*_*v*_ = 10.0, *ω*_*t*_ = 0.5 are weights to balance the significance of three objective components. Our results are not sensitive to the exact values of these weights, so other values within the same order of magnitude may be used as well.

### Objective Function for Stage II

The goal for the optimization in Stage II is to find the optimal values of synaptic weights in order to adapt to a variety of scenarios, specifically coping with curve trajectories and uneven terrains.

To accomplish this goal, we generate *N*_*s*_ samples from 2D uniform distribution of two variables: ${\theta }_{s}\in [-{\theta }_{max}^{s},{\theta }_{max}^{s}]$, ${\theta }_{t}\in [-{\theta }_{max}^{t},{\theta }_{max}^{t}]$ and enforce an objective function focusing on the trajectories of body center and foot:

15 $$f=\sum _{{N}_{s}}({\omega }_{s}\,{f}_{s}+{\omega }_{v}\,{f}_{v})$$

By summing up the errors of all samples, we consider the fitness of the individual solution under general scenarios.

## Results

### Implementation

**Figure 4:**
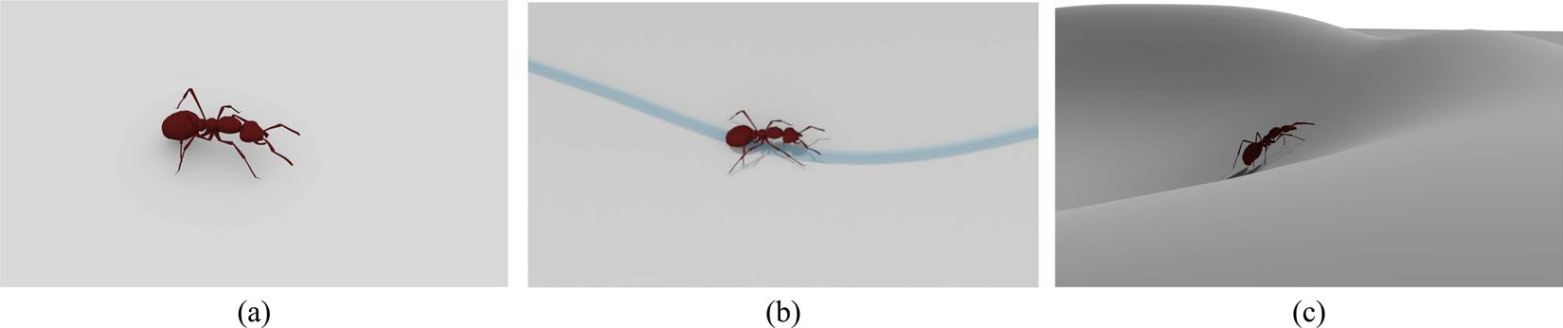
Screenshot of locomotion sequence of a virtual ant. (**a**) Walking straight forward. (**b**) Walking along a curve. (**c**) Walking on uneven terrain.

The code is written in C++ and runs on a standard PC with four CPU cores at 3.4 GHz. The physics simulation is done with the Bullet^31^ physics engine with a simulation step of 5 ms. Our single-thread implementation runs in real time during the online simulation, while the optimization process runs with the technique of multi-thread, costing around 10 hours for Stage I and 22 hours for Stage II on the aforementioned hardware. In comparison to the discretized look-up table^11^, our work provides a continuous mapping with comparable timecost. Our method also avoids manually selecting the value of the substep width which critically determines the quality and time performance of the look-up table method^11,32^. During the optimization, the simulation for each individual is run for 100 seconds (or 100 locomotion cycles) for Stage I, or 10 seconds (or 10 locomotion cycles) for Stage II, or is terminated in advance if the body falls down (the height of body center falls below a threshold). Figure 4 gives a screenshot of the locomotion sequence of a virtual ant in a variety of scenarios.

### Optimization

**Figure 5:**
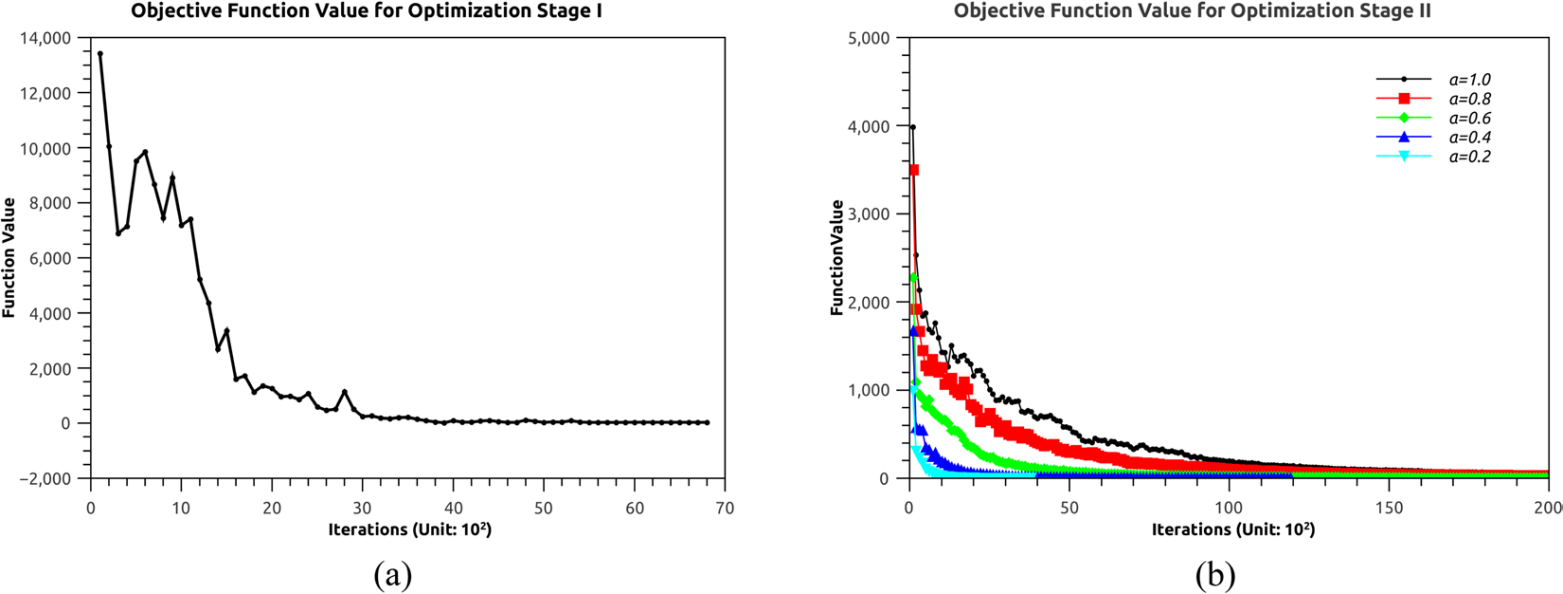
Objective function value of best individual from each generation for optimization Stage I (**a**) and Stage II (**b**).

The total dimensions of the parameter space is 11*18 = 198 for Stage I. For each generation, *λ* = 120 individuals are sampled from the elites and *μ* = 40 elites are selected out of the whole population. The values for the parameters for all neuron and muscle units are initialized as: *I* = 10 *mV*, *F*_*max*_ = 1, *β* = 1.5, *ρ* = 2.0, *ω* = 3.0, *V*_*max*_ = 5.0, *c*_*V*_ = −7.0, *b*_*V*_ = 0.67, *a*_*V*_ = −1.668. The triggering timing of the neuron models are set as *T*_*s*_ = 0,*T*_*e*_ = 0.5 for left front, right middle, left back legs, and *T*_*s*_ = 0.5, *T*_*e*_ = 1.0 for right front, left middle, right back legs. The success of the optimization is confirmed with the decreasing value of the objective function (Fig. 5). The objective function converges quickly in the first 2000 generations and slowly afterwards.

The total dimensions of the parameter space is 2*16 + 16*36 = 608 for Stage II. The values for the weight parameters are constrained as [0, 1]. The optimal values for the muscle parameters in Stage I (standard case of walking forward) are used at this stage. In practice, we found that it is difficult for the solution to converge given the high dimensions of the parameter space. To ensure the successful convergence, we initially sample the candidates from a narrower value range of [−$\alpha {\theta }_{max}^{s}$, $\alpha {\theta }_{max}^{s}$] and [−$\alpha {\theta }_{max}^{t}$, $\alpha {\theta }_{max}^{t}$], where *α* ∈ [0,1]. *α* starts from value of 0 and progressively increase to 1, with a step of 0.2, so that the initial value stays sufficiently close to the optimal value. Figure 5(b) shows the optimization progress for Stage II. The result shows that when the exploration range is small (for cases when *α* = 0.2 or 0.4), the optimization converges fast and smooth, while for other cases the optimization requires more iterations to converge.

### Spiking pattern and muscle activation

**Figure 6:**
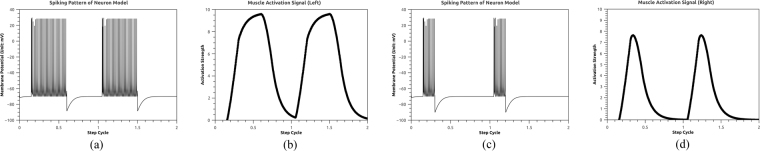
Spiking pattern and muscle activation of the neuron-muscle unit from the body-coxa joint on the left-sided and right-sided middle leg, when the character turns left. (**a**) Spiking pattern on the left leg. (**b**) Activation signal on the left leg. (**c**) Spiking pattern on the right leg. (**d**) Activation signal on the right leg.

Figure 6 plots the spiking pattern and muscle activation signals of the retractor neuron-muscle unit from the body-coxa joint on the left-sided and right-sided middle legs, when the character turns left. With the activation dynamics (Equation 6), the discrete spiking signal is converted into a continuous activation stimulus. The result shows that when the character turns left, the number of spike trains on the left-sided leg is greater than the one on the right-sided leg. This leads to a larger activation signal for the muscle actuation. The result also shows that the coordination between different legs is achieved by preferably adjusting the number of spike trains, instead of the amplitude of the spike.

### Comparison of joint trajectory between the real and virtual ants

**Figure 7:**
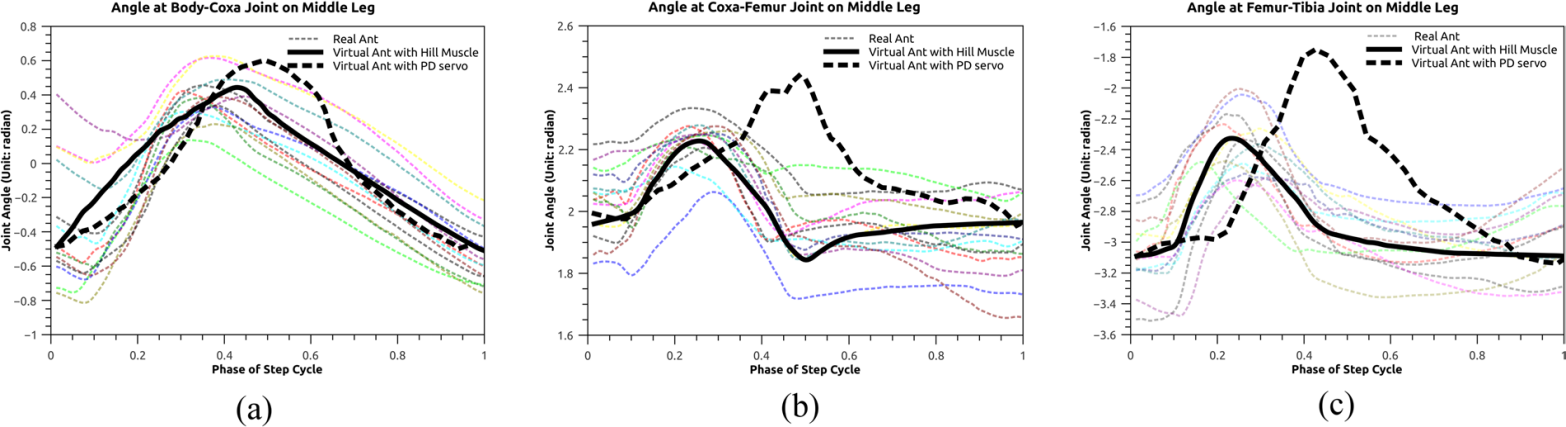
Joint angles on the middle leg captured from real insects during a locomotion cycle. Dashed lines are trajectories from real ants, solid line (in bold) is from virtual ant with Hill-type muscle and dashed line (in bold) is from virtual ant with PD servo.

One of the objective functions is to minimize the difference between the trajectory of the real and virtual insects. We here compare the joint angles at three DOFs on the middle leg for both the real and simulated subjects (Fig. 7). We found that the overall shape of the trajectory is consistent between the real and simulated cases. However in the result of real cases, the joint angles decrease to a minor extent before increasing incrementally. This is not observed in the simulated case, in particular for the body-coxa joint. This mismatch may be caused by the posture adjustment of real ants, or possibly by the hardware limitation (image resolution and framerate) of data collection. In the simulation case, the body-coxa joints move at a strict forward pattern in a single motion cycle.

### Comparison of joint torque between the real and virtual ants

**Figure 8:**
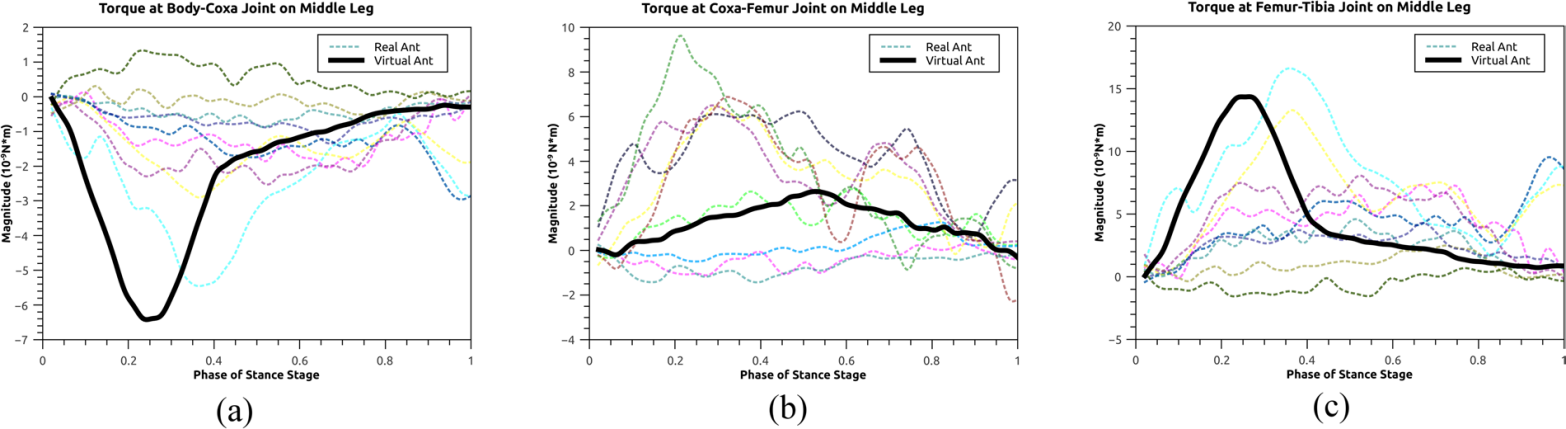
Joint torques on the middle leg during the stance stage in the mode of walking straight forward.

**Figure 9:**
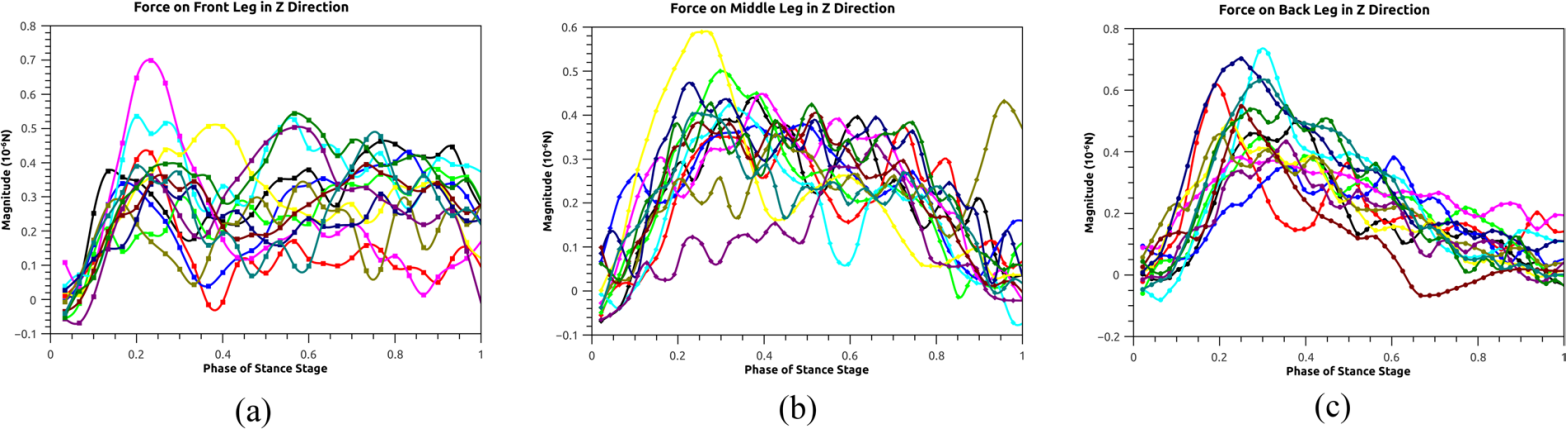
Ground forces in the Z direction during the stance stage in the mode of walking straight forward.

Figure 8 plots the joint torques on the middle leg during the stance stage for both the simulated and real cases. The torque from the simulation cases are recorded as the torques applied on the joint constraints during the simulation step. The torque from the real cases are computed with the method of Virtual Model Control^11^. This method is based on Jacobian matrix, converting the ground force on the end-effector into the joint torques given the configuration of body hierarchy. The peak of the simulation torques arises in the first half of the stance stage for the body-coxa and femur-tibia joints, and in the middle of the stance stage for the coxa-femur leg. However, the muscle activation in the simulation case reaches its peak around the end of the stance stage, instead of the first half. This is possibly due to the fact that the joint rotation reaches its limit at the end of the stance stage, resulting in a small value of *F*_*L*_ and *F*_*V*_ (Equation 9) and thus a moderate value of joint torque. The torque profiles of real cases generally follow the shape of a parabolic curve and have the peak value around the middle of the stance stage. This is consistent with the ground force on the middle leg (Fig. 9(b)), which presents a similar parabolic curve shape.

Figure 9 plots the ground forces on the real ant in the Z (vertical up) direction during the stance stage. For the front leg, the force magnitude present small variations during the whole stance stage. For the middle leg, the force magnitude increases initially, remains stable and decreases when the stance stage comes to the end. This creates a parabolic curve of the force profile. For the back leg, it presents a distinct peak during the first half of the stance stage, which is in consistent with the torque results in the simulation case.

## Discussion

### Advantages of spiking neuron model

Replicating the function of neural system in real organism is non-trivial, given the fact that the machinery underlying the observed behaviour is not fully understood^32^. Existing works have proposed different paradigms to model the behaviour neural system. For example, previous work uses a network of nonlinear oscillator to construct the Central Pattern Generator^11^. We believe the use of different models has its own pros and cons. The oscillator with inherent properties of limit cycle and attraction point could generate the basic pattern with no intervention from higher control system. The high-level neural system is encoded as a look-up table^11^, which is pre-computed and discretized. In comparison, the current work, which is based on spiking neuron models, provides a unified representation of neural system. The spiking pattern of the unit neurons can be used to seamlessly connect the procedures of sensory processing, high-level commands and low-level control strategy. Such a unified representation lays the foundation for processing sensory information via large-scale networks.

### Advantages of Hill-type muscle

The use of nonlinear Hill-type muscle improves the naturalness of the synthetic motion on the examples of biped^28^, and research also show that it improves the 2D motion stability for insect simulation^33^. However the significance of non-linear muscle has not been fully evaluated in the example of a 3D insect model. We here compare the performance of two actuator choices: Proportional-derivative (PD) servo^11^ and nonlinear Hill-type muscle, in terms of the naturalness of the synthetic motion (Fig. 7). The PD servo is defined as:

16 $$\tau ={k}_{g}({q}_{t+1}-{q}_{t})+{k}_{d}{\dot{q}}_{t}$$

${q}_{t+1},{q}_{t},{\dot{q}}_{t}$ are the rotational angles and velocity of the joint. *k*_*g*_, *k*_*d*_ are the gain and damping coefficients.

To quantitatively evaluate the difference between the ground-truth data and the simulated trajectories, we use the Euclidean distance between two vectors:

17 $$d=||{{\bf{T}}}^{g}-{{\bf{T}}}^{s}||$$

where ${{\bf{T}}}^{g}=[{T}_{1}^{g},{T}_{2}^{g},\cdots {T}_{n}^{g}],{{\bf{T}}}^{s}=[{T}_{1}^{s},{T}_{2}^{s},\cdots {T}_{n}^{s}]$ are joint trajectories of rotation in one step cycle from the ground truth and simulated result respectively. **T**^*g*^ is computed as the average of all collected trajectories of real ants.

The quantitative result of comparison (Table 3) shows that trajectories of our model presents less deviations from the ground-truth data than the model in^11^. This is mainly caused by the linear feature of the PD servo. We also notice that the peak values of the trajectories of the previous model^11^ are generally higher than the ones of the proposed model in this work. The reason is possibly caused by the fact of over-shooting^34^. The problem of over-shooting refers to the fact that the PD servos normally require a large value of *k*_*g*_, thus causing the over-protraction/elevation/extension of the foot.

**Table 3:** Quantitative comparison of the trajectory difference between the ground-truth data and the ones generated by our model and the previous model^11^.

Joints	Our Model	Previous Model^11^
body-coxa	0.7188	1.4827
coxa-femur	0.3467	1.7306
femur-tibia	0.4962	4.1081

The reason is possibly caused by the fact of over-shooting^34^. The problem of over-shooting refers to the fact that the PD servos normally require a large value of *k*_*g*_, thus causing the over-protraction/elevation/extension of the foot.

The neuro-musculo-skeletal model we developed yields very promising results. The CMA optimization algorithm successfully helps to find optimal parameter configuration for the model. The optimization process converges quickly in around 2000 iteration. And the simulated gait pattern is highly consistent with the experimental data collected from real ants walking in the free mode.

An interesting and promising point is that the joint torques of the simulated and real cases also present high consistency. It will be a great help to biologist in studying of some special phenomenons of insects such as the incredible lifting ability of ants and impressive leaping ability of mantis. They can easily observe and analysis the stress conditions under various situations with the simulated model. And the study can stimulate the development of bionic design.

The model could also be beneficial in studying of higher level behavior of insects. With the model, we can reproduce the motion of an insect with only the recorded foot trajectories. Thus, we can use techniques of machine learning to extrapolate or predict the trajectories of insects under different circumstances and then reconstruct the 3d motion with our model. Our model is intended to be widely applicable in its ability to model insect locomotions. Here we only conduct a primary validation on ants. It will be interesting to apply this model to other insect species in future, potentially including other locomotion constraints.

In conclusion, we have proposed a neuro-musculo-skeletal model of virtual insects. By incorporating some nature-tested mechanisms of real insects, the model is capable of reproducing the gait pattern observed from real insects on virtual ones. There are a couple of directions for future works. The current implementation uses a single neuron to simulate the neuron group activating the same muscle. Improving the model complexity to match the real insect is worth exploring. To extend this framework to these advanced motion skills is one of the future efforts. How to encode more sophisticated behaviours, such as collective transport^35^, as patterns of spiking trains is even more challenging.

## Electronic supplementary material

Supplementary Information

Supplementary Video
