# Supplementary material for: A Neuro-Musculo-Skeletal Model for Insects With Data-driven Optimization
Source: Sci Rep. 2018 Feb 1;8:2129. doi: 10.1038/s41598-018-20093-x (PMC5795013; doi:10.1038/s41598-018-20093-x)
Supplement: Supplementary file 1 — Supplementary Information [file 41598_2018_20093_MOESM1_ESM.pdf]

Manuscript Title

A Neuro-Musculo-Skeletal Model for Insects With Data-driven Optimization

Author List

Shihui Guo
Juncong Lin
Toni Wöhrl
Minghong Liao

Video Caption

Supplementary video for the manuscript "A Neuro-Musculo-Skeletal Model for Insects With Data-driven Optimization"
